# PTPIP51: A New Interaction Partner of the Insulin Receptor and PKA in Adipose Tissue

**DOI:** 10.1155/2013/476240

**Published:** 2013-03-06

**Authors:** M. A. Bobrich, S. A. Schwabe, A. Brobeil, M. Viard, M. Kamm, F. C. Mooren, K. Krüger, C. Tag, M. Wimmer

**Affiliations:** ^1^Institute of Anatomy and Cell Biology, Justus Liebig University Giessen, Aulweg 123, 35392 Giessen, Germany; ^2^Department of Neurology, Justus Liebig University Giessen, 35392 Giessen, Germany; ^3^Department of Sports Medicine, Justus Liebig University Giessen, 35394 Giessen, Germany

## Abstract

*Aims*. Our previous experiments revealed an association of PTPIP51 (protein tyrosine phosphatase interacting protein 51) with the insulin signalling pathway through PTP1B and 14-3-3beta. We aimed to clarify the role of PTPIP51 in adipocyte metabolism. *Methods*. Four groups of ten C57Bl/6 mice each were used. Two groups were fed a standard diet; two groups were fed a high-fat diet. Two groups (one high-fat diet and one standard diet) were submitted to endurance training, while the remaining two groups served as untrained control groups. After ten weeks, we measured glucose tolerance of the mice. Adipose tissue samples were analyzed by immunofluorescence and Duolink proximity ligation assay to quantify interactions of PTPIP51 with either insulin receptor (IR) or PKA. *Results*. PTPIP51 and the IR and PTPIP51 and PKA, respectively, were colocalized in all groups. Standard diet animals that were submitted to endurance training showed low PTPIP51-IR and PTPIP51-PKA interactions. The interaction levels of both the IR and PKA differed between the feeding and training groups. *Conclusion*. PTPIP51 might serve as a linking protein in adipocyte metabolism by connecting the IR-triggered lipogenesis with the PKA-dependent lipolysis. PTPIP51 interacts with both proteins, therefore being a potential gateway for the cooperation of both pathways.

## 1. Introduction

Obesity abets several life style diseases such as diabetes type II, atherosclerosis, or impaired wound healing. These adverse effects can be avoided or, if already present, reduced by physical activity. Physical activity increases sympathetic activity and blood catecholamine levels. Adaption to periodic physical exercise is well studied. Exercise training and increased catecholamine levels affect adipocyte metabolism, on one hand by influencing insulin secretion [1] and on the other hand by additionally channelling the adipocyte metabolism towards lipolysis [2]. This effect is mediated by beta2-adrenergic receptors which activate the intracellular adenylyl cyclase. Adenylyl cyclase transforms ATP into cyclic AMP (cAMP), which in turn increases the activity level of cAMP-dependent protein kinase A (PKA) [3, 4]. Protein kinase A thereupon initiates lipolysis [4].

The most potent antagonist of catecholamines on adipose tissue is insulin. Activation of the insulin receptor results in decreased lipolysis, increased lipogenesis and uptake of fatty acids. 

Lipolysis is decreased by insulin-receptor-induced phosphodiesterase-3B (PDE-3B) and 14-3-3beta-dependent reduction of PKA activity [3, 5]. Furthermore, the insulin receptor activates several lipogenic enzymes by tyrosine phosphorylation and promotes GLUT-4 recruitment to the cell membrane [6]. 

Protein tyrosine phosphatase interacting protein 51 (PTPIP51) is expressed in different organs with a multitude of functions [7, 8]. As previously shown for mouse adipose tissue, PTPIP51 acts as an effector in insulin signalling. PTPIP51 expression was correlated to the insulin resistance of the animals [9], according to the expression of its interaction partner protein tyrosine phosphatase 1B (PTP1B). In addition to that, PTPIP51 is connected to the lipolytic MAPK pathway by its interaction with raf-1 mediated by 14-3-3beta [7]. 14-3-3beta also interacts with phosphodiesterase 3B, resulting in reduced intracellular cAMP levels and decreased PKA activity [9]. As 14-3-3beta and PTP1B are important for adipocyte metabolism control, the role of its interaction partner PTPIP51 needs to be disclosed. 

The interaction of PTPIP51 with the insulin receptor and PKA was investigated in adipose tissue from animals kept under different defined experimental conditions to search for a potential role of PTPIP51 in adipocyte metabolism. 

## 2. Material and Methods

### 2.1. Study Design

The experiments were performed with male C57Bl/6 mice (*n* = 40) and approved by the local Animal Care and Use Committee (Gi 20/24 Nr. 94/2010). The animals were kept under standard conditions (12 h light/dark cycle) with no access to running wheels. They were fed ad libitum with free access to water. The animals were aged ten weeks at the beginning of the training. The experiments were run for 14 weeks, with a four-week period of fat feeding followed by a ten-week training period. 

Control group (SD): the animals were fed a standard diet (Altromin standard diet no. 1324, Altromin, Lage, Germany). 

Endurance training control group (SDT): the animals were fed a standard diet (Altromin standard diet no. 1324, Altromin, Lage, Germany) and trained for a period of ten weeks. The training was performed on a motorized treadmill for 35 min/day at 12 m/min, at 12% grade, 5 times per week, for ten weeks. Running speed was 0.27 m/s ± 0.05 m/s corresponding to 80% of maximal oxygen consumption.

High-fat-diet group (HFD): the animals were fed a high-fat diet containing 45% fat for 14 weeks of the experimental period.

High-fat-diet, training group (HFDT): The animals were fed a high-fat-diet during the complete experimental time period. The endurance training period started four weeks after the beginning of high-fat-diet feeding and was similar to the training described above.

For diet composition, see Table 1. 

After 14 weeks, the animals were sacrificed in the morning between 10 and 12 o'clock and the abdominal adipose tissue was frozen in liquid nitrogen precooled isopentan, and transferred to −80°C till further analysis. See also Bobrich et al. [9]. A glucose tolerance test was performed to determine the insulin sensitivity of the mice [9–11]. For testing the glucose tolerance of the animals, they were injected intraperitoneally with 2 g of 20% D-glucose dissolved in sterile 0.9% NaCl solution per kg body weight. The blood was collected and glucose concentration was determined. The test was performed in the morning; last feeding of the animals took place the previous day.

### 2.2. PTPIP51 Antibody Production

An antibody against a defined peptide sequence at the C-terminus of PTPIP51 (sequence: IQKDLEELEVILRD, exon 13) was produced (BioLux, Stuttgart, Germany). Identity and purity of the synthesized peptide were approved by ESI-MS and UV-analysis. Rabbits were immunized with the KLH-conjugated peptide. The specificity of the antibody was tested by ELISA and Western blot. Preabsorption experiments were performed [9, 12].

### 2.3. Immunofluorescence

Immunofluorescence staining was performed according to a standard protocol [9]. The primary PTPIP51 antibody to the C-terminus was used in 1 : 800 dilutions and visualized by Alexa 555 (Molecular Probes, Darmstadt, Germany, Cat. no. A21428). Primary monoclonal mouse antibodies were used for double staining experiments with PKA (ab58187, Abcam plc, 330 Cambridge Science Park, Cambridge, UK) and the beta-subunit of the IR (clone CT-3, Millipore, 28820 Single Oak Drive, Temecula, CA, USA). To avoid unspecific binding of the primary mouse antibodies, samples were preincubated with biotin-coupled anti-mouse-antibodies for one hour. The reaction of the primary monoclonal mouse antibody was visualized using Alexa fluor 488 secondary antibodies (Molecular Probes, Darmstadt, Germany, Cat. no. A11001).

### 2.4. Confocal Laser Scanning Microscopy

Confocal images of cells were obtained with a Leica confocal laser scanning microscope (CLSM, 5 TCS SP2, Leica, Bensheim, Germany). Confocal images of Alexa Fluor 555 fluorescence were acquired using 6 Plan-Apochromat  × 40/1.4 oil objective, 548 nm excitation wavelengths (helium-neon laser), and a 560–585 nm band-pass filter. The pinhole diameter was set to yield optical sections of 1 airy unit. For the detection of Alexa Fluor 488, we used a Plan-Apochromat × 40/1.4 oil objective, the 488 nm excitation wavelength of an argon laser, and a 505–530 nm band-pass filter. The pinhole diameter was set to yield optical sections of 1 Airy unit. Confocal images of To-Pro-3 fluorescence (Molecular probes, Cat. no. T3605) (nuclear staining) were acquired using Plan-Apochromat × 40/1.4 oil objective, 633 nm excitation wavelengths (helium-neon laser), and the 650–670 nm bandpass filter. The pinhole diameter was set to yield optical sections of 1 Airy unit. Acquired DIC and confocal images were analyzed and combined using the LCS software (Leica Confocal Software). 

Acquired images were subsequently processed by ImageJ (v1.43 m; Rasband, W.S., ImageJ, US National Institutes of Health, Bethesda, MD, USA, http://imagej.nih.gov/ij/, 1997–2011) using an iterative deconvolution plug-in by Bob Dougherty (http://www.optinav.com/imagej.html, Iterative Deconvolution). Options were set for all confocal acquired images as follows: 8 numbers of iteration and 2.0 pixels of LP filter diameter. Point spread function was calculated for each channel separately by the ImageJ plug-in created by Bob Dougherty (http://www.optinav.com/imagej.html, Diffraction Limit PSF).

### 2.5. Intensity Correlation Analysis

Intensity correlation analysis (ICA) was carried out using ImageJ (v1.43 m; Rasband, W.S., ImageJ, US National Institutes of Health, Bethesda, MD, USA, http://imagej.nih.gov/ij/, 1997–2011) and an appropriate plug-in for ICA included in the plug-in package of the Wright cell imaging facility [13] (http://www.uhnres.utoronto.ca/facilities/wcif/fdownload.html).

### 2.6. Duolink Proximity Ligation Assay (DPLA)

Interaction of PTPIP51 with either PKA or beta-subunit of the IR was detected by the proximity ligation assay kit Duolink (Olink *|*Bioscience, Uppsala, Sweden, PLA probe anti-rabbit minus for the detection of the rabbit PTPIP51 antibody, Cat. no. 90602; PLA probe anti-mouse PLUS for the detection of the mouse anti PKA or IR antibody, Cat. no. 90701; Detection Kit 563, Cat. no. 90134). 

**Figure 1:**
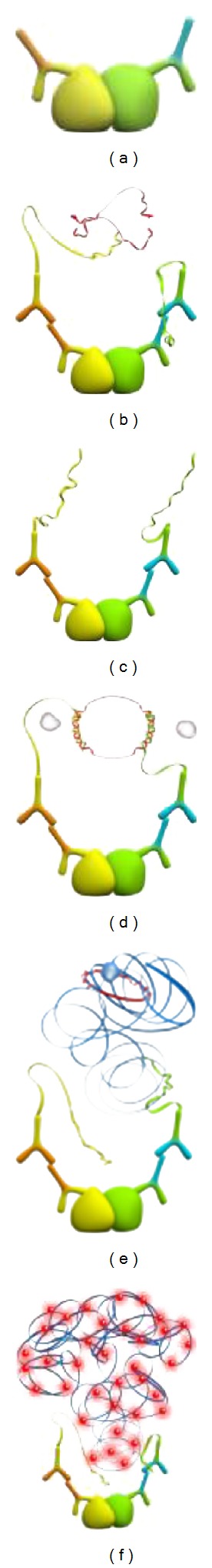
The principle of the Duolink proximity ligation assay. (a) Binding of primary antibodies to PTPIP51 and the IR or PKA. (b) Binding of secondary antibodies, PLA probe PLUS and PLA probe MINUS. (c) Connector oligos only hybridize if both proteins are closer than 40 nm. (d) Ligation of the complex forms a circular template. (e) Rolling circle amplification. (f) Amplification yields a some hundredfold replication, and added fluorophore labelled probes highlight the reaction, resulting in a point-shaped signal. Figure 1 is a reproduction of the Olink instruction manual by courtsey of Olink Bioscience.

The DPLA was performed according to the manufacturer's protocol; negative controls were included [9, 14, 15]. For the principle of the DPLA, see Figure 1.

### 2.7. Fluorescence Microscopy

The Axioplan 2 fluorescence microscope equipped with Plan-Apochromat objectives (Carl Zeiss Jena, Germany) was used for photo documentation. For visualization of the DPLA, an excitation filter with a spectrum of 530–560 nm and an emission filter with a spectrum of 572–647 nm were used. 

### 2.8. Analysis of Duolink Proximity Ligation Assay Results

The Duolink Image Tool (Olink biosciences, Uppsala, Sweden) was used for quantification of the detected DPLA signals. All analyzed sections were investigated with equal light intensity and shutter speed. Sensitivity of the software scan and blob threshold were set identically for all probes. The positive reaction dots were counted automatically. 

### 2.9. Database Research

We accomplished a database research using the GPS 2.1 database [16] to identify kinases within adipocyte metabolism that possess the ability to phosphorylate PTPIP51. 

### 2.10. Statistical Analysis

The observed data were analysed by nonparametrical tests to determine the significance of the results.

## 3. Results

### 3.1. Glucose Tolerance Test and Mean Body Weight

The glucose tolerance was determined by a standard glucose tolerance test at the end of the experimental period. SD and SDT animals displayed a slight increase of blood glucose levels 30 minutes after application of glucose which was almost normalized after 60 minutes. 

In HFD animals, blood glucose levels strongly increased from the basal level of 100 mg/dL to 400 mg/dL 60 minutes after application. Blood glucose levels did not return to normal values within 120 minutes.

**Figure 2:**
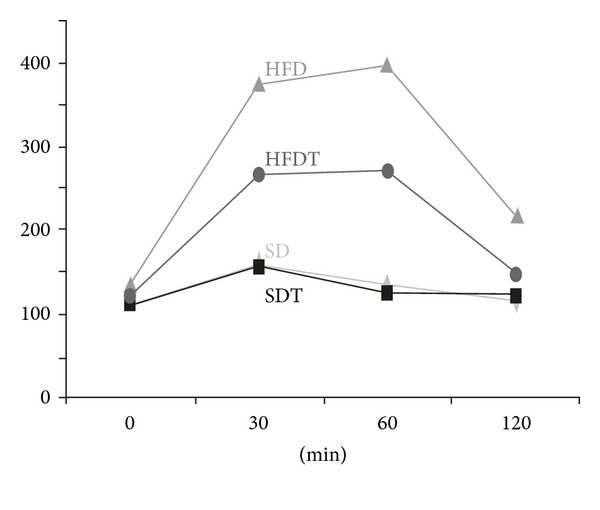
Glucose tolerance test: average of blood glucose concentrations in mg/dL after intraperitoneal application of glucose solution. Blood glucose levels were determined 0, 30, 60, and 120 minutes after application. Glucose tolerance is inversely related to insulin resistance. SD standard diet group (*n* = 10), SDT standard diet and training group (*n* = 10), HFD: high-fat-diet group (*n* = 10), HFDT: high-fat-diet and training group (*n* = 10).

In HFDT animals, blood glucose levels increased to 270 mg/dL 30 minutes after application and almost returned to normal values after 120 minutes (Figure 2). The values of HFD, HFDT, and animals fed a standard diet (SD and SDT) are significantly different after 30 and 60 minutes. After 120 minutes, the differences between standard diet and high fat diet animals remained significant, whereas there was no significant difference between standard diet and trained high-fat-diet animals. 

Mean body weight was measured at different time points throughout the test (see Table 2). 

Statistical analysis showed that there was no significant difference between the four groups at 10 weeks of age. From the beginning of the training, the difference between standard diet and high-fat-diet-fed groups was highly significant, but there was no significant difference between the SD and SDT and, respectively HFD and HFDT groups. 

### 3.2. PTPIP51 Protein Is Colocalized with the Insulin Receptor (IR) and Protein Kinase A (PKA)

**Figure 3:**
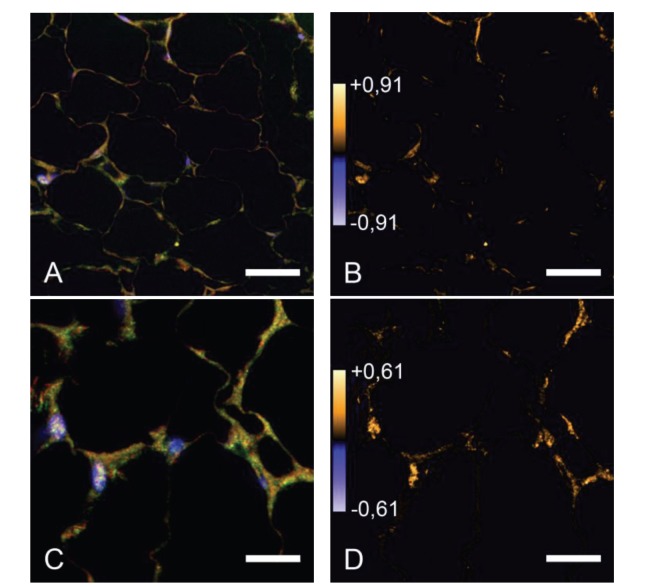
Immunostaining of PTPIP51, insulin receptor, and PKA in adipocytes of standard diet animals. (A) Immunostaining of PTPIP51 and the insulin receptor. (B) Intensity correlation of PTPIP51 and the insulin receptor, high correlation is displayed in yellow. (C) Immunostaining of PTPIP51 and PKA. (D) Intensity correlation of PTPIP51 and the insulin receptor, high correlation is displayed in yellow; Bar (A): 60 *μ*m; Bar (C): 15 *μ*m.

PTPIP51 localization in adipose tissue did not differ between the four experimental setups. PTPIP51 immunoreactivity was detected within the cytoplasm and at the plasma membrane (Figure 3). 

The IR revealed a similar expression pattern when compared to PTPIP51. In the cytoplasm, the immune reaction of the IR antibody was lower compared to PTPIP51. The computed data of the intensity correlation analysis is displayed in Figure 3(B). Colocalization is displayed in yellow to orange and noncolocalized parts are shown in blue. Most colocalization was detected at the plasma membrane. 

PKA is also colocalized with PTPIP51, especially concerning its expression in the cytoplasm. The computed data of the intensity correlation analysis is displayed in Figure 3(D). Co-localization is displayed in yellow to orange and non-co-localized parts are shown in blue. Most colocalization was detected within the cytoplasm.

### 3.3. PTPIP51 and IR Interact and the Interaction Depends on the Feeding Status

**Figure 4:**
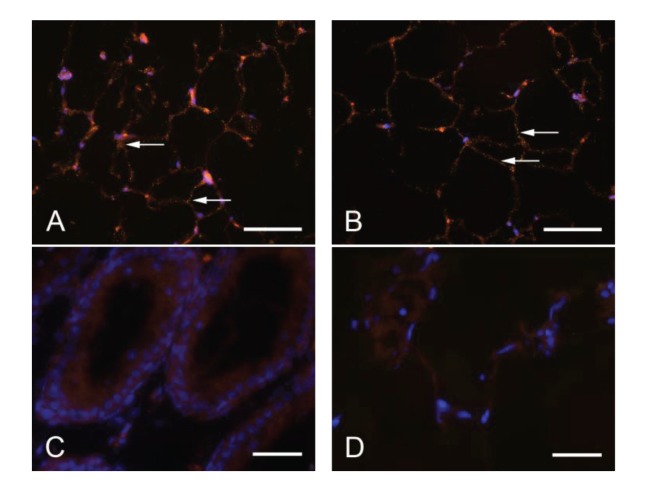
Duolink proximity ligation assay to detect interactions between PTPIP51 and the insulin receptor (IR), respectively PTPIP51 and PKA in standard diet animals. (A) Interaction between PTPIP51 and the IR. (B) Interaction between PTPIP51 and PKA. (C) Negative control, PTPIP51 and the IR in testis, no interaction known. (D) Negative control, DPLA without primary antibodies in adipose tissue. Nuclei are marked by DAPI staining. Arrows: positive reactions. Bars (A, B): 50 *μ*m. Bars (C, D): 20 *μ*m.

The Duolink proximity ligation assay (DPLA) was performed to detect interactions between PTPIP51 and the beta-subunit of the IR. The interaction of PTPIP51 and IR was verified as seen in Figure 4, where each dot indicates the interaction of both proteins. This interaction of PTPIP51 and the IR was found in the adipocytes of animals from all experimental groups. Specificity of the DPLA was tested (Figure 4).

**Figure 5:**
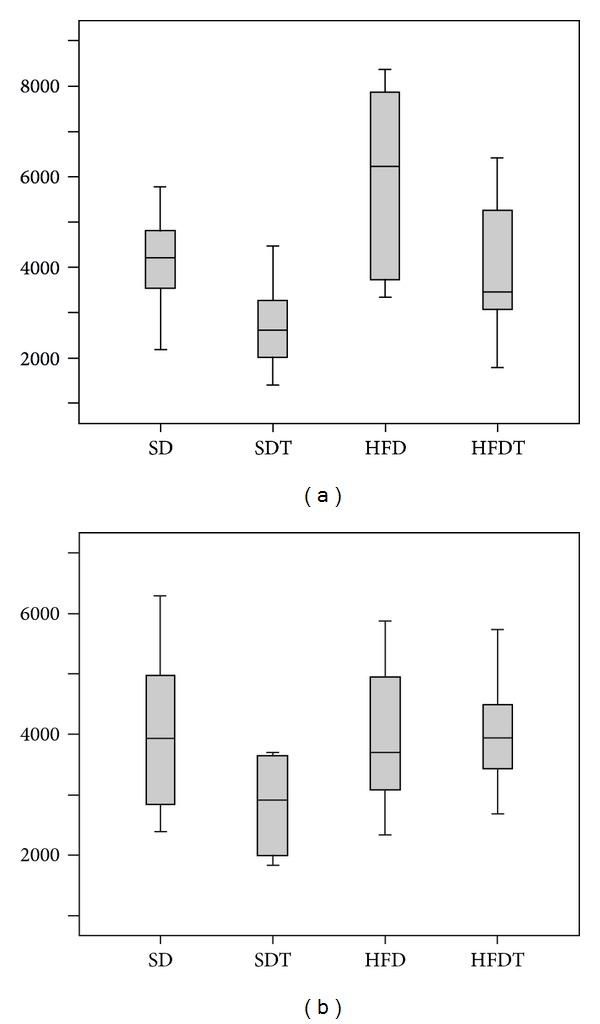
Semiquantitative analysis of the interaction between PTPIP51 and its interaction with IR and with PKA. Every section was subdivided into rectangles of the same size and dots were counted. The results were averaged for each section. (a) Semiquantitative evaluation of PTPIP51 interaction with IR. (b) Semiquantitative evaluation of PTPIP51 interaction with PKA. SD: standard diet group. SDT: standard diet and training group. HFD: high-fat-diet group. HFDT: high-fat-diet and training group.

The grade of interaction in adipose samples from SD animals corresponds to the level in HFDT animals. A decrease in the interaction was detected in samples from SDT animals. HFD animals displayed the highest interaction level (Figure 5(a)).

### 3.4. Interaction between PTPIP51 and PKA Displayed a Dependency on Training Status

The Duolink proximity ligation assay was performed to detect interactions between PTPIP51 and PKA. The assumption of an interaction of PTPIP51 and PKA based on the database research was verified (Figure 4). PTPIP51 and PKA were interacting in adipocytes of animals from all experimental groups.

The interaction levels of SD, HFD, and HFDT mice did not show significant differences. The interaction levels seen in SDT mice were decreased (Figure 5(b)). 

## 4. Discussion

To sum up the results, adipocytes from mice subjected to different diets and activity levels displayed a connection between PTPIP51 and the insulin receptor, and PTPIP51 and PKA respectively. Animals of the four experimental groups displayed differences in their glucose resistance. The glucose-tolerance test revealed a glucose-intolerant status in HFD and HFDT animals, whereas the glucose tolerance of HFDT animals improved compared to HFD animals. 

Immunofluorescence data displayed colocalization of PTPIP51 with IR and PKA suggesting an interaction between PTPIP51 and IR, and PTPIP51 and PKA respectively. Basing on these facts, we performed a GPS 2.1 database research. Search results displayed PTPIP51 to be phosphorylated at tyrosine 176 by the insulin receptor and at serine 46 by PKA [16]. These two phosphorylation sites are possible regulatory sites for the interaction of PTPIP51 with 14-3-3beta, thus being able to influence the MAPK pathway by interaction with raf-1 [17]. Phosphorylation of Serine 46 in PTPIP51 results in augmented interaction with raf-1 via 14-3-3 and thus in increased ERK1/2 phosphorylation, promoting the MAPK pathway [12, 17]. The MAPK pathway is suspected to possess two opposite functions in adipocytes. Depending on the activator, ERK1/2 conveys either adipocyte growth or lipolysis [18]. PKA is known to induce lipolysis through the MAPK pathway [18, 19]. 

The insulin receptor induces tyrosine phosphorylation of PTPIP51 at tyrosine 176, which leads to reduced interaction of PTPIP51 with 14-3-3beta and raf-1 [20], thereby decreasing the MAPK pathway activity.

Analysis of the PTPIP51 interaction profile of adipocytes displayed high interaction levels with the insulin receptor in HFD animals. Interaction levels were reduced by 30% in SD and HFDT and by 50% in SDT animals, compared to HFD animals. 

High-fat feeding increases the lipogenic activity in adipose tissue [21, 22]. This is consistent with our observation of high interaction levels in HFD animals. Training leads to reduced adipocyte size [23]. The reduced PTPIP51-IR interaction in trained groups compared to the corresponding untrained groups suggests that PTPIP51 is especially involved in the processes of insulin induced lipogenesis or in its antilipolytic action.

Interaction levels between PTPIP51 and PKA did not show significant differences in SD, HFD, and HFDT animals. In SDT animals, the interaction was reduced. 

The low interaction in SDT animals seems to be controversial. But, as PKA also interacts with the hormone-sensitive lipase (HSL), a change of the interaction partner of PKA away from PTPIP51 under these preconditions is assumable. The interaction between PKA and HSL is a potent promoter for lipolysis in adipocytes. The activities of both PKA and HSL are increased in endurance-trained animals [24, 25]. 

Our experiments suggest that PTPIP51 could mediate between lipogenesis and lipolysis by switching between the lipogenic insulin pathway and the lipolytic PKA pathway. Further experiments need to be conducted in order to prove our hypothesis and to determine the magnitude in which PTPIP51 engages in adipocyte metabolism.

## Figures and Tables

**Table 1:** Energy content of standard diet and high-fat diet.

Values in kcal/100 g	Standard diet	High-fat diet
Protein	24	20
Carbohydrates	65	35
Fat	11	45

**Table 2:** Mean body weight in grams with standard deviation.

	Basic value (10 weeks of age)	Beginning of training (14 weeks of age)	End of test period (24 weeks of age)
Standard diet (SD)	23.22 ± 2.41	23.16 ± 1.03	27.74 ± 0.96
Standard diet and training (SDT)	22.81 ± 3.11	22.79 ± 0.77	26.54 ± 1.15
High fat diet (HFD)	23.67 ± 3.02	40.37 ± 2.97	50.91 ± 2.56
High fat diet and training (HFDT)	23.44 ± 2.88	42.22 ± 3.18	49.23 ± 3.43

## Abbreviations

PKA: cAMP-activated protein kinase A
DPLA: Duolink proximity ligation assay
HFD: High fat-diet
HFDT: High-fat diet and training
HSL: Hormone-sensitive lipase
IR: Insulin receptor
MAPK: Mitogen-activated protein kinase
PDE-3B: Phosphodiesterase-3B
PTPIP51: Protein tyrosine phosphatase interacting protein 51
SD: Standard diet
SDT: Standard diet and training.
